# Supplementary material for: Characterization of the Molecular Events Underlying the Establishment of Axillary Meristem Region in Pepper
Source: Int J Mol Sci. 2023 Aug 12;24(16):12718. doi: 10.3390/ijms241612718 (PMC10454251; doi:10.3390/ijms241612718)
Supplement: Supplementary file 1 [file ijms-24-12718-s001.zip › Table S2.pdf]

Supplementary materials

Table S2 Summary of RNA sequencing and assembly

Samples	Obtained Reads	OBtained Base(bp)	Q30(%)	GC(%)
A1-1	22,304,832	6,674,142,204	94.05	43.05
A1-2	26,023,912	7,788,307,930	93.94	43.09
A1-3	22,823,203	6,830,831,808	93.7	43.16
A-2-1	23,668,282	7,083,093,858	94.23	43.11
A-2-2	22,031,770	6,593,950,342	93.65	43.1
A-2-3	22,539,220	6,743,956,116	94.07	43.09
A-3-1	20,510,645	6,139,776,564	93.17	42.95
A-3-2	22,429,753	6,711,745,054	93.17	42.97
A-3-3	22,770,448	6,806,687,212	93.41	43.05
B-1-1	23,110,379	6,915,045,410	94.11	44.07
B-1-2	21,235,798	6,355,300,496	94.13	44.07
B-1-3	20,858,612	6,242,490,374	94.06	44.28
B-2-1	19,756,888	5,912,551,984	93.72	44.1
B-2-2	20,837,014	6,235,898,274	94.28	44.07
B-2-3	22,786,667	6,819,108,570	94.19	43.99
B-3-1	19,300,909	5,776,282,088	93.6	43.67
B-3-2	19,988,758	5,977,828,252	93.51	43.99
B-3-3	20,321,939	6,078,738,230	94.03	44.12
C-1-1	20,277,503	6,064,779,048	91.12	42.67
C-1-2	19,961,269	5,970,335,780	90.90	43.10
C-1-3	23,149,176	6,926,575,290	92.89	44.39
C-2-1	20,574,635	6,157,927,870	94.12	43.87
C-2-2	25,959,397	7,768,225,360	94.07	43.92
C-2-3	19,882,232	5,950,499,140	93.74	43.92
C-3-1	19,648,251	5,878,764,034	93.76	43.95
C-3-2	20,399,873	6,104,784,178	93.15	43.87
C-3-3	20,067,188	6,004,192,140	93.43	43.8
