# Supplementary figures and images for: Characterization of the Molecular Events Underlying the Establishment of Axillary Meristem Region in Pepper
Source: Int J Mol Sci. 2023 Aug 12;24(16):12718. doi: 10.3390/ijms241612718 (PMC10454251; doi:10.3390/ijms241612718)

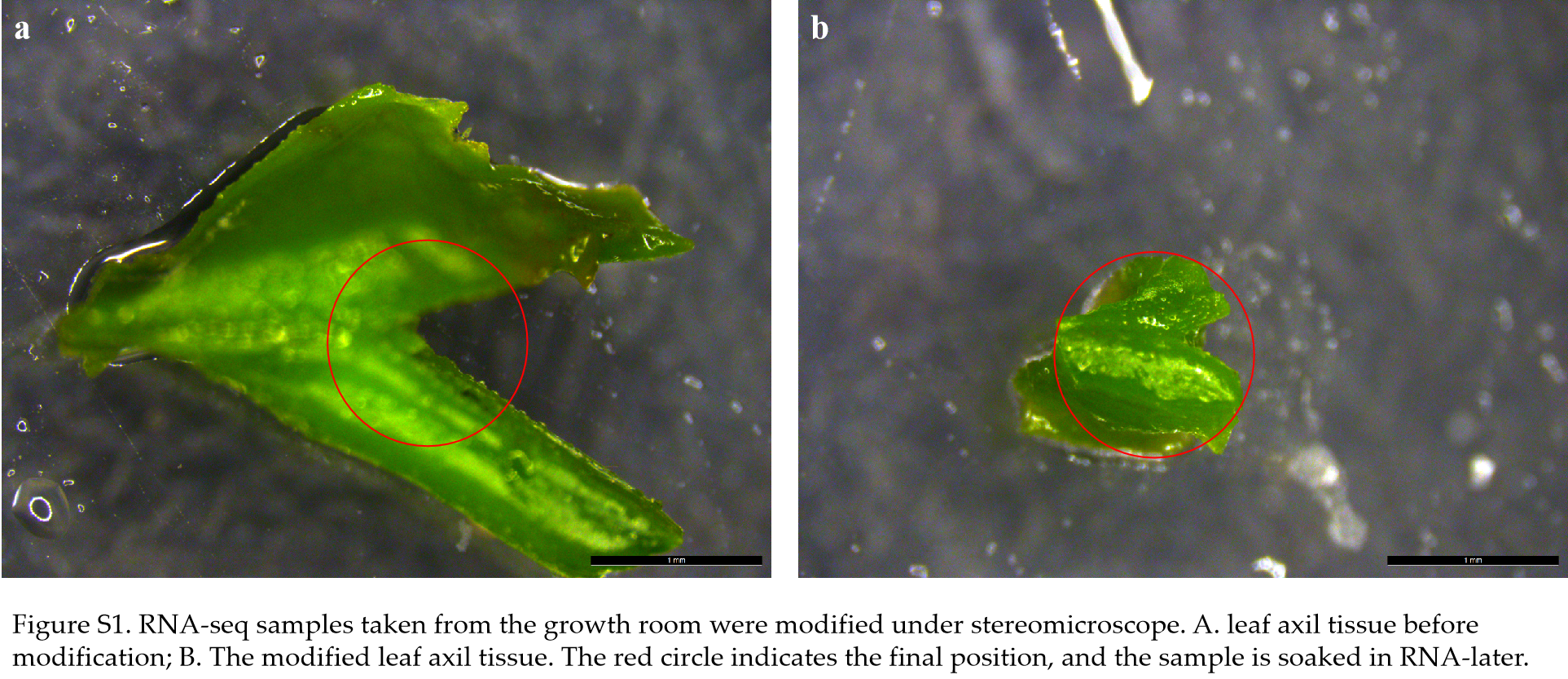

Supplement: Supplementary file 1 [file ijms-24-12718-s001.zip › Figure S1.png]

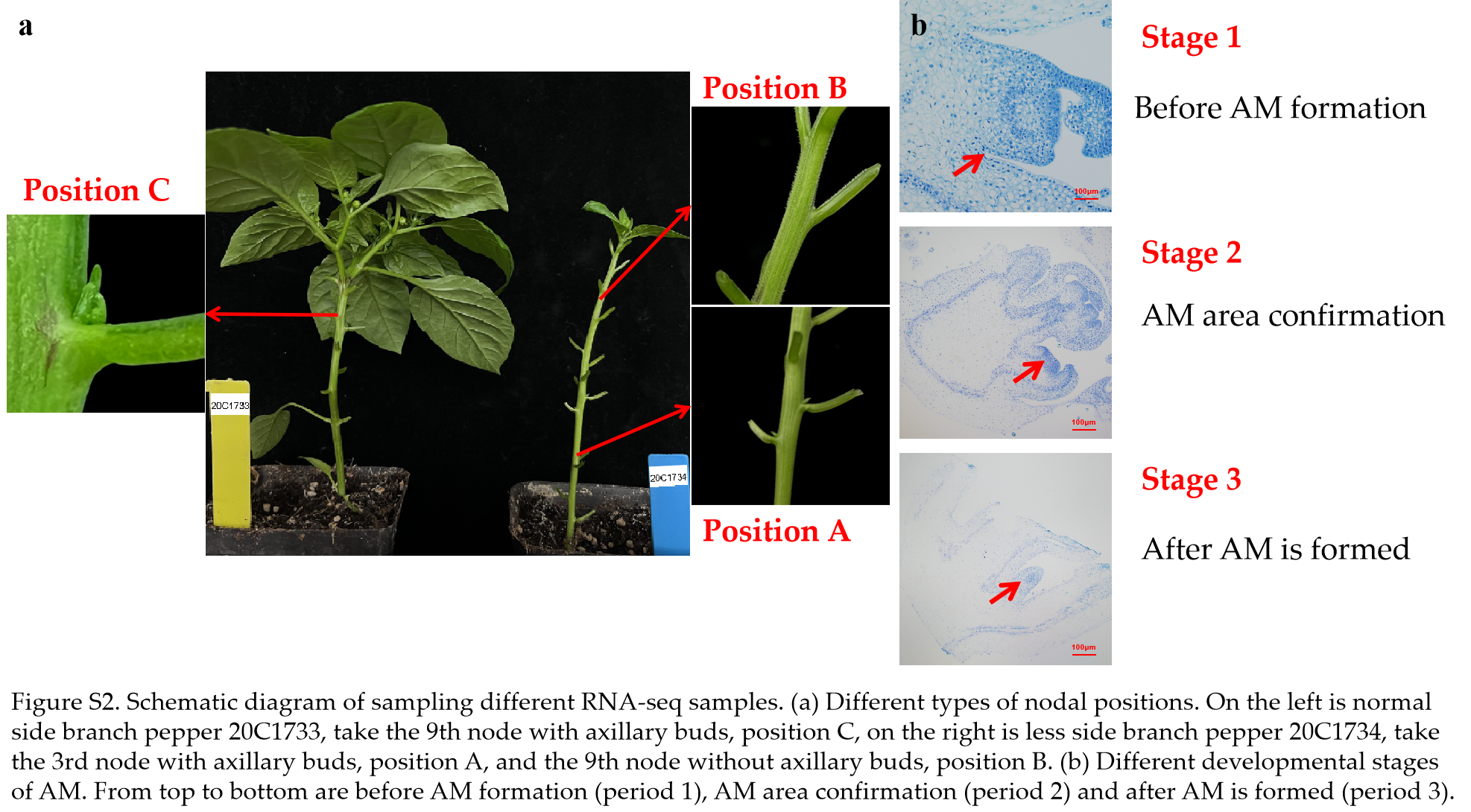

Supplement: Supplementary file 1 [file ijms-24-12718-s001.zip › Figure S2.tif]

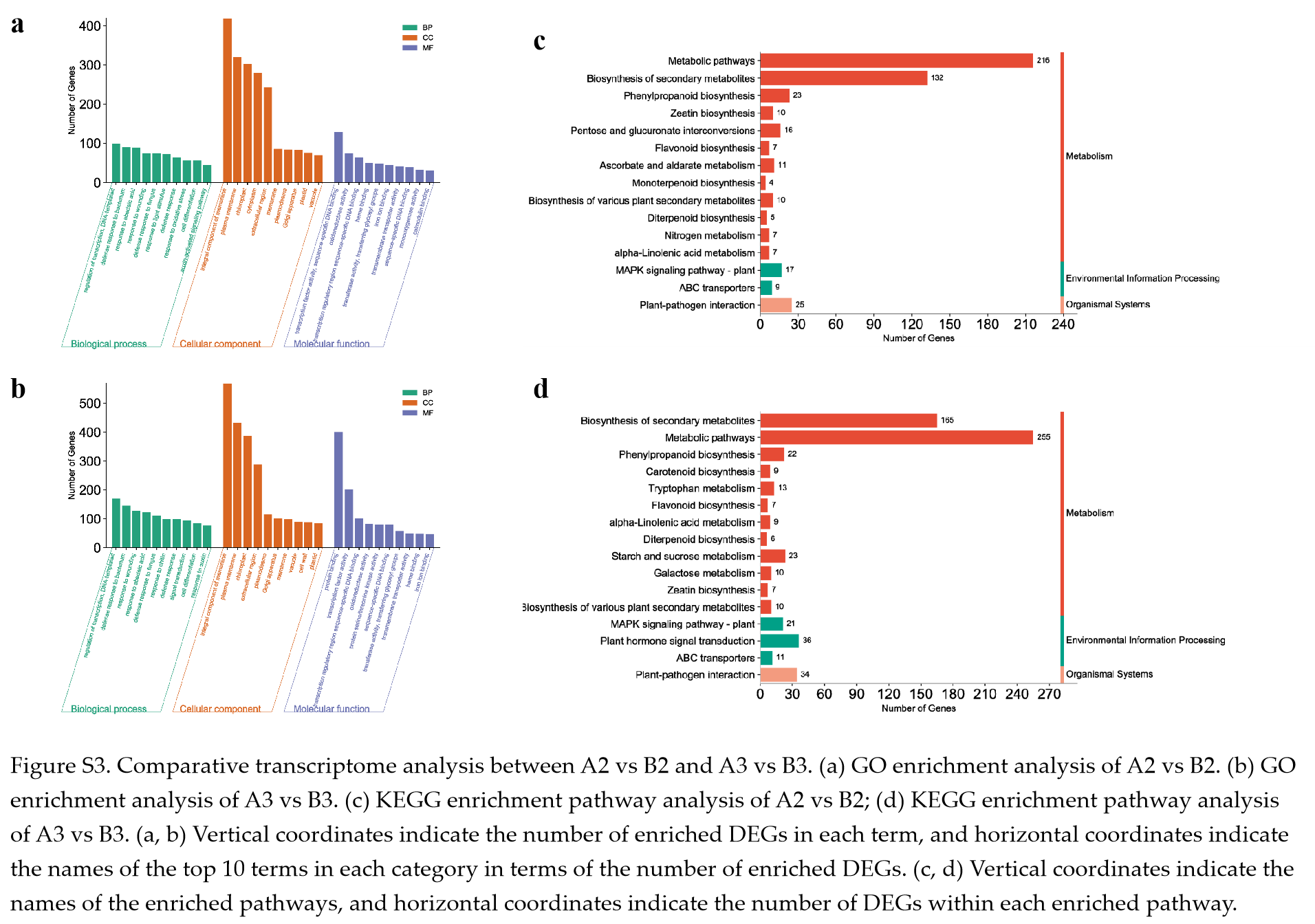

Supplement: Supplementary file 1 [file ijms-24-12718-s001.zip › Figure S3.png]

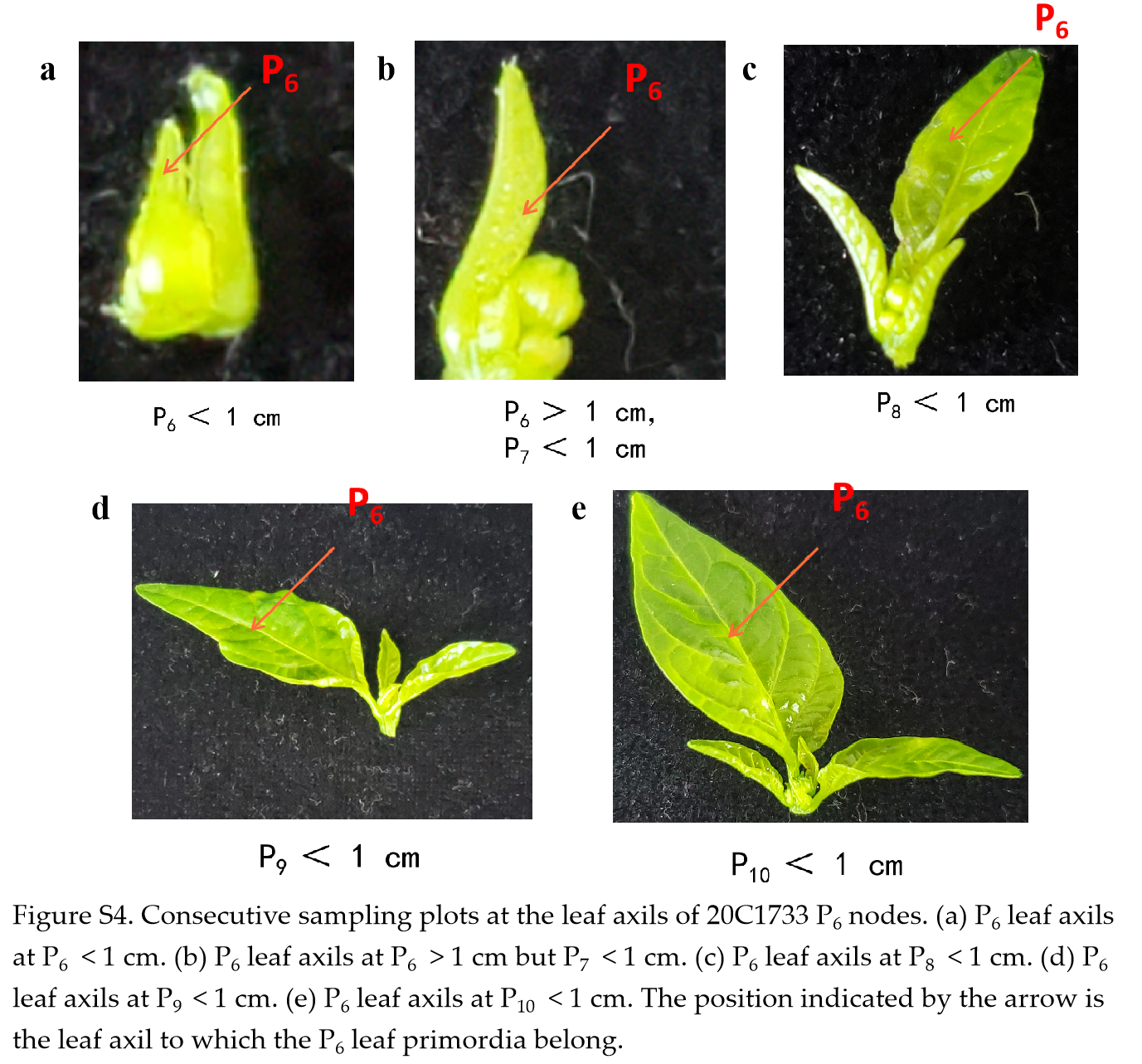

Supplement: Supplementary file 1 [file ijms-24-12718-s001.zip › Figure S4.png]
